# Vaccination and Smallpox

**Published:** 1899-03

**Authors:** 


### VACCINATION AND SMALLPOX.

IN view of the fact that there is now in the United States considerable smallpox it is in order to call the attention of physicians and the people to the necessity of thorough vaccination, as the only preventive. There is yet in the civilized world, even in England the home of Jenner, much prejudice against vaccination. But when it comes to a choice between the disease and the prophylactic, most objections to vaccination vanish.

Vaccine points are unreliable and subject to contamination. The tubes of glycerinated vaccine are sterile and keep so. The results obtained are in every way more satisfactory. In experiments with the denudation method (*Hutchins*) the vaccine used from tubes was productive of the best results.

Statistics show that three to four points of inoculation afford a better protection than one or two. The denudation method is better than scarification, because it is without pain to the subject, and strong men have fainted under the scarification method, the denudation can be as rapidly done, chances of extra infection are lessened and there is no oozing of blood to bar the absorption of the virus.

Many persons have been "vaccinated," got a sore which was only an infection with pus, or other organisms, had a terrible "time" and thought they were finely protected, when in fact they were never really vaccinated at all. It is hinted that a few cases of smallpox have recently appeared here, though the newspapers have said nothing; and it is the duty of doctors to look out for it. Every person should be protected by vaccination, properly done, whether there is any outbreak of smallpox or not.

---

IT is now just a little over a month before the annual meeting of the Georgia Medical Association. The Secretary of the Association and the Chairman of the Program Committee, Dr. Barron of Macon, have already sent out to the members circular letters, urging upon them to be present and to aid in making the meeting a grand success. As we have already before stated in an editorial, this meeting of the Association will mark the fifty years of its existence and therefore will be one of more than usual interest. The Association meets in Macon and we are informed that the physicians in that city are making great preparations to entertain in royal style, and those of us who have been to Macon before, know that things are never done but in the most approved style. We would urge upon physicians all over the State to begin to make their arrangements now, as far as possible, in order that they may be present and also prepare themselves to read a paper. We say "prepare themselves" advisedly, because we do not believe in physicians reading papers before a large body of intelligent physicians without preparation beforehand in order that they may not appear themselves to a disadvantage and at the same time show no familiarity with the subject. Let us all make this meeting of the Association the best one in its history.

On another page of the JOURNAL will be found a communication from the President of the Association, Dr. Howard Williams, who gives us in brief some of the good things which will occur. Every indication points to a most excellent meeting, and now let every member of the Association do his part.

Let the next meeting of our Association be well attended by the physicians all over the State. We should have as members of this body all the regular physicians in the State. Let us make a suggestion to those who intend to read a paper. Make your papers practical and have them within the limits. There are very few men who can read a paper longer than twenty minutes and at the same time hold the attention of his auditors. Bring out the salient features of your article in a few words, but make them terse. The President wishes us to call attention to Article No. VIII, of the By-Laws and for the benefit of our readers we will publish it in full:

PAPERS.

SECTION 1. The titles of all papers to be read at any annual meeting shall be furnished the Chairman of the Program Committee thirty days before the meeting.

SEC. 2. Not more than twenty minutes shall be occupied in reading any paper. No speaker shall be allowed to speak a second time on the same subject, nor consume more than five minutes in the discussion.

SEC. 3. No paper shall occupy over twenty pages in the Transactions.

SEC. 4. The Publication Committee shall decline to publish any paper not handed the Secretary complete before the final adjournment of the annual meeting.

SEC. 5. No paper shall be read before the Association that has already been published or that has been read before any other body.

SEC. 6. A member presenting a paper must be present at the meeting in order to have it published in the Transactions.

---

WE have just received the most brazen-faced circular from Lincoln, Neb., that was perhaps ever received by a physician supposed to have two grains of sense. Surely our friend in Nebraska must imagine that we are the most gullible set of fools who ever drank milk in our infanthood.

The circular is dated Lincoln, Neb., and starts off with this heading: Better than a Post Course on Rectal Diseases, Hernia and Catarrh. Stay at home and learn how to cure these diseases in your office. Why go away to see what your patients would not let you do when you get home?

Following this the gentleman tells you in confidence that he has made so much money out of this new treatment which he has devised, that he wants you to become rich also.

One of the questions asked is: "Are you making as much money as you ought to make?" This implies that he is, but out of solicitation for your welfare, he is willing to sacrifice himself to help you make more money. However, you will find that this is not all gratuitious philanthropy on his part, as after expatiating on the great material wealth which will be yours by using his remedies, he adds simply as a corollary, "For the small fee of $15.00, I will give you in printed pamphlet form, the formulæ with complete directions for injections, operations, applications and treatment in specialties. I now include with my work the formula for the Woman's Specific. It is the best combination of remedies for most non-surgical diseases of women. It would add very much to your success in this line. I also give the best treatment for gonorrhœa. It would make your treatment of this disease a pleasure."

At the end of his circular the names of several gentlemen who live in Lincoln are added who will vouch for his standing in the community, but adds, "I obtained the names of these parties with the understanding that they were not to be called upon to answer inquiries. If they were open to correspondence, they would be overwhelmed with letters to no purpose at all, for they could only say over in substance what they have already said."

---

It is with pleasure that we can call attention to two reminiscent medical articles by two of our older physicians. Dr. Rae, of Alabama, has given us an excellent, practical article, and which clearly shows that fifty years ago "advanced ideas" were used. Dr. G. G. Roy gives us another article from his early experiences in the practice of medicine, which will prove of interest to all, but especially to the older practitioners. We wish we could have more of this kind. Can't some of our old physicians, who are readers of THE JOURNAL, give us some of their early experiences?

---

THE January issue of the *American Journal of Surgery and Gynecology* is a Woman's Number. As such, it is of course, "red hot." This is a distinct departure for medical journalism, although not an uncommon occurrence in the secular press. Usually the proceeds of such an edition are given to some charitable object, but failing to see such an announcement, we suppose we will find it appearing in the February number. We congratulate the lady editor and her associates, but we believe the invitations called for "no flowers."

---

DR. E. R. ANTHONY, Secretary of the State Board of Medical Examiners, informs us that the exact date for holding the next meeting for examination of candidates has not been definitely decided, but in all probability it will be held in Atlanta between the 1st and 3d of April, and immediately afterward in Augusta, Ga. Dr. W. A. O'Daniel is now President of the Board.

---

WE wish to call attention to an error which occurred in the last issue of THE JOURNAL. Dr. Patterson's article should have read "Rational Surgery in Cross-Eyes" instead of "Retinal Surgery."

---

THE Atlanta College of Physicians and Surgeons will hold its Commencement at the Grand Opera House on Monday evening, April 3d. Colonel N. J. Hammond will make the address of the occasion. The college under its new management will have a large graduating class.

---

WE call attention to the special spring course of the Chicago Policlinic and Hospital. See ad., p. xxiv.

---

***NOTICE.—This JOURNAL and THE INTERNATIONAL JOURNAL OF SURGERY one year for $1.00 cash, to new subscribers. No out-of-town checks accepted unless 10c. is added for cost of cashing.***

